# Association between dietary *β*-carotene intake with Parkinson’s disease and all-cause mortality among American adults aged 40 and older (NHANES 2001–2018)

**DOI:** 10.3389/fnut.2024.1430605

**Published:** 2024-09-30

**Authors:** Jing Su, Liming Liu, Ruonan Wang, Chunmei Li, Zihan Wang, Qiaoli Xu, Chunyu Shen, Dalong Wu, Dexi Zhao

**Affiliations:** ^1^College of Chinese Medicine, Changchun University of Chinese Medicine, Changchun, China; ^2^Department of Encephalopathy, The Affiliated Hospital of Changchun University of Chinese Medicine, Changchun, China; ^3^College of Nursing, Changchun University of Chinese Medicine, Changchun, China; ^4^Changchun University of Chinese Medicine, Changchun, China

**Keywords:** dietary *β*-carotene intake, Parkinson’s disease, all-cause mortality, National Health and Nutrition Examination Survey, cross-sectional study, cohort study

## Abstract

**Background:**

The existing evidence concerning the correlation between dietary *β*-carotene intake and Parkinson’s disease (PD) is currently deemed insufficient. Thus, this research aims to investigate the relationship between dietary *β*-carotene intake and both the prevalence of PD and all-cause mortality within the US (United States) population.

**Methods:**

The research employed cross-sectional analysis and cohort studies utilizing data from 16,852 participants in the National Health and Nutrition Examination Survey (NHANES) spanning from 2001 to 2018. Weighted logistic regression, weighted cox regression, restricted cubic splines (RCS), subgroup analysis, and sensitivity analyses were employed to validate the research objectives.

**Results:**

Among all eligible subjects, the mean age was 59.62 ± 11.77 years, with a prevalence of PD at 1.82% overall, with 43.88% in males. In the fully adjusted model, dietary *β*-carotene intake exhibited a negative association with PD prevalence [odds ratio (OR) = 0.95; 95% confidence interval (CI): 0.90 ~ 0.997; *p* = 0.040]. Utilizing RCS analysis, a negative linear correlation between dietary *β*-carotene intake and PD prevalence was observed (non-linear *p* = 0.857). Furthermore, after controlling for multiple variables, dietary *β*-carotene intake was inversely associated with all-cause mortality [Hazard ratios (HR) = 0.98; 95% CI: 0.97 ~ 0.99; *p* = 0.002], with RCS curves indicating a negative linear relationship (nonlinear: *p* = 0.082). Comparable patterns of association were noted in subgroup analyses, and consistent findings were derived from additional sensitivity analyses.

**Conclusion:**

The cross-sectional and cohort study reveals a significant negative correlation between dietary *β*-carotene intake and both the prevalence of PD and all-cause mortality in the general population. This suggested that supplementing with dietary *β*-carotene might have certain benefits for reducing the prevalence of PD and all-cause mortality. However, further rigorously designed expected studies are needed to establish the causal relationship between them.

## 1 Introduction

PD, a chronic central nervous system disorder often referred to as shaking palsy, is considered the second most prevalent neurodegenerative disease globally, significantly impacting patients’ lives and health (1). From 1990 to 2015, the number of PD patients doubled to over 6 million (2). By 2040, the prevalence of PD is estimated to rise from 6.9 million individuals in 2015 to 14.2 million, making it a significant future global health challenge (3). Despite the availability of some treatment methods, there is currently no specific therapy to effectively halt the progression of PD, with only symptomatic relief medications prescribed (4). Therefore, exploring risk factors and therapeutic strategies for preventing and delaying disease progression are crucial. One of PD’s hallmark features is the sustained and specific degeneration or demise of dopamine neurons within the brain, emphasizing the importance of preserving these neurons for PD treatment (5). Excessive accumulation of reactive oxygen species, often stemming from mitochondrial abnormalities or inflammation, emerges as a primary aspect leading to dopaminergic neuron degeneration in PD, providing potential therapeutic targets (6). Additionally, neuroinflammation and oxidative stress represent significant factors implicated in damaging dopamine receptors, thus accelerating the degenerative process of dopaminergic neurons (7). Oxidative stress’s contribution is a critical factor in the complex cascade of neurodegenerative events, promoting the degeneration of dopaminergic neurons and exacerbating PD’s pathological progression (8, 9). Consequently, antioxidants play a beneficial role in preventing and managing PD by reducing oxidative stress and damage to neurons (10–12). Recent studies suggest that consuming antioxidant-rich foods may alleviate PD symptoms and slow its progression, offering new avenues for developing novel treatment strategies (13–18).

*β*-carotene, a natural antioxidant, is classified as a non-polar carotenoid commonly found in various fruits and vegetables, imparting them with red or orange pigments, such as carrots, sweet potatoes, and pumpkins (19). Research has elucidated the multifaceted roles of *β*-carotene within the human body. Apart from its well-established antioxidant properties, it exhibits anti-apoptotic, anti-aging, and neurodevelopmental regulatory functions (19–21). These diverse mechanisms underscore the potential health benefits associated with *β*-carotene consumption. Moreover, emerging evidence suggests a plausible connection between *β*-carotene and neurodegenerative diseases, including cognitive decline, PD, and Alzheimer’s disease, underscoring its relevance in maintaining neurological health (17, 22–26). Recent studies utilizing the NHANES database have reported an inverse correlation between increased dietary *β*-carotene intake and the risk of cognitive decline in elderly individuals (27). These findings not only corroborate the neuroprotective effects of *β*-carotene but also warrant further investigation into its potential therapeutic applications in neurodegenerative disorders.

Additionally, comprehensive exploration of the relationship between *β*-carotene concentrations and PD risk in the U.S. population remains scarce. Thus, this study aimed to bridge this gap by investigating the potential involvement of dietary *β*-carotene intake in PD among American adults through a cross-sectional study in NHANES. Simultaneously, the study assessed the impact of dietary *β*-carotene intake on all-cause mortality in the general population through a cohort study.

## 2 Methods

### 2.1 Study population and research methods

**Figure 1:**
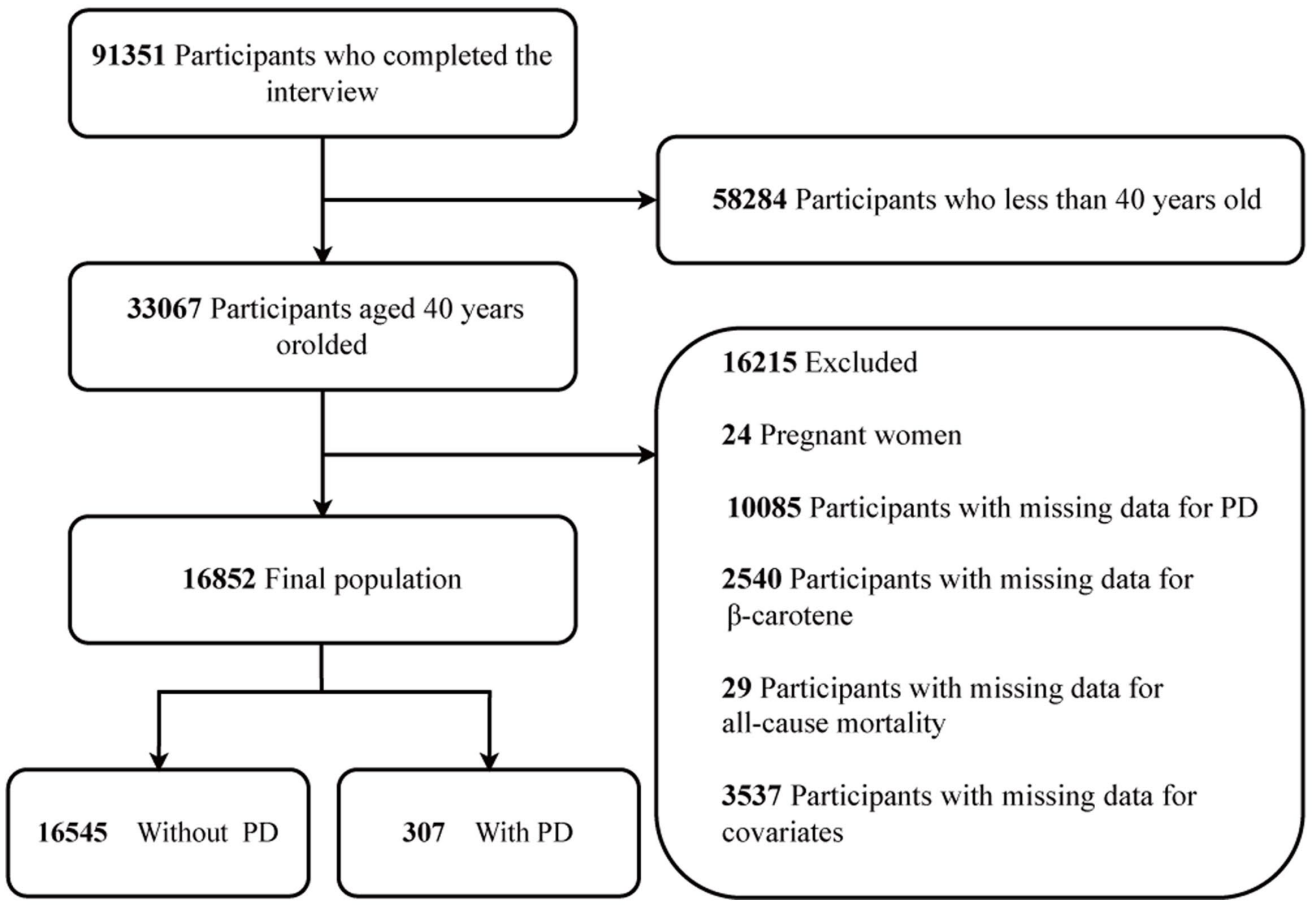
Flowchart depicting the selection strategy. Exegesis: PD, Parkinson’s disease.

The NHANES website provides access to all survey data. Conducted on a two-year cycle, NHANES is a comprehensive study of the US population, employing a multi-stage and stratified sampling design to ensure national representativeness (28). For this study, a total of 91,351 subjects were selected from 9 consecutive NHANES survey cycles spanning from 2001 to 2018. To ensure age uniformity, those under 40 years old (*n* = 58,284) were excluded. Moreover, individuals who were pregnant (*n* = 24), had missing data for PD (*n* = 10,085), lacked data on *β*-carotene (*n* = 2,540), or had missing data for all-cause mortality (*n* = 29) were also excluded. Additionally, 3,537 study individuals were excluded owing to missing data on other variables. Consequently, the final study population comprised 16,852 participants. The recruitment process is depicted in Figure 1.

A cross-sectional study was used to investigate the relationship between dietary *β*-carotene intake and the prevalence of Parkinson’s disease. A cohort study was employed to explore the association between dietary *β*-carotene intake and all-cause mortality.

### 2.2 Measurement of the dietary *β*-carotene intake

The dietary interview section of the NHANES was executed through a collaborative effort between the U.S. Department of Agriculture and the Department of Health and Human Services, overseen by the National Center for Health Statistics (NCHS), which managed both the survey sample design and data collection processes. The methodology employed in dietary assessment, formulated by the Food Surveys Research Group, was instrumental in ensuring the maintenance of standardized investigation protocols and stringent quality controls throughout all phases of the survey administration.

In prior research, data concerning *β*-carotene intake was acquired through 24-h dietary recall interviews (27, 29). The initial dietary recall session occurred in person during the visit, followed by a ensuing interview implemented via telephone within 3 to 10 days. Comprehensive information on the dietary data collection process is available on the NHANES website. Individuals provided detailed information regarding various food and beverage items, contributing to the nutrient intake profiles. Data regarding recalls were sourced from the “Total Nutrient Intakes” files. In this research, *β*-carotene intakes were derived from data obtained during the first day of dietary interviews in the Total Nutrient Intakes dataset.

### 2.3 Diagnosis of PD

In this research, PD as the outcome variable. Individuals diagnosed with PD were identified by categorizing prescriptions under the “Second Level Category Name” as “ANTIPARKINSON AGENTS” in the Prescription Medications document. This determination relied on participants’ responses to inquiries regarding prescribed medications. Due to the constraints of medications and codes contained in NHANES, the classification of PD required individuals to be actively undergoing treatment for the condition. Individuals who did not disclose the usage of anti-parkinsonian medication were classified as non-PD participants. The disease definition utilized in this study aligns with criteria established in previous research (30).

### 2.4 Assessment of mortality

The NCHS utilizes mortality data sourced from the National Death Index, cross-referencing various demographic attributes including social security numbers, names, dates of birth, race/ethnicity, gender, birth status, and residential status (31). Supplementary materials for mortality follow-up include the US Social Security Administration, the Centers for Medicare and Medicaid Services, and death certificates. Participants enrolled in the NHANES from 2001 to 2018 were prospectively monitored from their enrollment date until December 31, 2019 (32). This comprehensive approach ensures a thorough understanding of mortality trends and outcomes among the surveyed population, contributing valuable insights for public health interventions and policy formulation.

### 2.5 Measurements of other covariates

Drawing from existing literature and clinical practice, we assessed a range of potential covariates (30, 33), encompassing sociodemographic information, lifestyle variables and comorbidities. Structured data collection in NHANES encompasses certain sociodemographic information. Participants provided self-reports on age, sex, race/ethnicity (Non-Hispanic White, Non-Hispanic Black, Mexican American, Other Hispanic, and Other Race), marital status (married or cohabiting with a partner, or living independently), family poverty income ratio(≤ 1.3, 1.3 to 3.5, > 3.5) and education (less than high school, high school or equivalent, and above high school) (34). Following definitions found in previous literature, smoking status was classified as follows: never smokers (<100 cigarettes in their lifetime), current smokers (>100 cigarettes in life and smoke some days or every day), and former smokers (>100 cigarettes in their lifetime but currently do not smoke at all) (35). Self-reported drinking status was classified as follows: never drinkers (<12 drinks in their lifetime), former drinkers (consumed ≥12 drinks in 1 year but did not drink in the last year, or did not drink in the last year but consumed ≥12 drinks in their lifetime), mild drinkers (females ≤1 and males ≤2 drinks/day), moderate drinkers (females ≤2 and males ≤3 drinks/day), or heavy drinkers (females ≥3 and males ≥4 drinks/day) (28). Trained health technologists conducted measurements of weight and height in accordance with the anthropometry procedure manual. Subsequently, BMI was computed by dividing weight in kilograms by height squared in meters. Both coronary heart disease and stroke were self-reported by participants. In calculating the average blood pressure for hypertension, the following protocol was followed (35): (1) Diastolic readings of zero were excluded from the calculation of the diastolic average. (2) If all diastolic readings registered as zero, the average was documented as zero. (3) If only one blood pressure reading was obtained, that single measurement was considered as the average. (4) In cases where multiple blood pressure readings were available, the initial reading was consistently excluded from the average calculation. The diagnostic criteria for diabetes encompass the following indicators: (1) Diagnosed with diabetes by a doctor. (2) Glycohemoglobin HbA1c(%) > = 6.5. (3) Fasting glucose (mmol/l) > = 7.0. (4) Random blood glucose (mmol/l) > = 11.1. (5) Two-hour OGTT blood glucose (mmol/l) > = 11.1. (6) Utilization of diabetes medication or insulin (35). The data on dietary vitamin E intake, dietary vitamin C intake, dietary copper intake, dietary iron intake, and dietary niacin intake were obtained through 24-h dietary recall interviews (27, 29).

### 2.6 Statistical analyses

In our analysis, due consideration was given to the intricate sampling design and sample weights for dietary intake (WTDRD1). Normally distributed continuous variables were reported as mean ± standard deviation (SD), while categorical variables were depicted as frequencies and percentages. Distinctions among groups were assessed using the chi-square test for categorical variables, One-Way Analysis of Variance for normally distributed variables, and the Kruskal-Wallis H test for variables displaying a skewed distribution. The impact of dietary *β*-carotene intake on PD was evaluated using binary weighted logistic regression models, presenting OR and 95% CI. Cox proportional hazards models were employed to compute HR and corresponding 95% CIs for all-cause mortality associated with dietary *β*-carotene intake and PD. Additionally, Kaplan–Meier (KM) survival analysis was conducted to examine the all-cause mortality rate based on tertiles of dietary *β*-carotene intake and PD status, with assessment facilitated through the log-rank test.

We chose these confounders based on clinical relevance, previous scientific literature (36). We constructed 5 models: Model 1 adjusted for no covariate adjustment. Model 2 was further accounted for age, sex, race/ethnicity (demographic factors). Model 3 additionally controlled for marital status, education level, family income (socioeconomic factors). Model 4 further adjusted for BMI, smoking status, drinking status (lifestyle factors). Model 5 (main model) additionally considered coronary heart disease, stroke, hypertension, diabetes (disease history).

We utilized RCS models to generate smooth curves, allowing us to investigate potential nonlinear dose–response relationships between *β*-carotene and both PD and all-cause mortality. In this model, *β*-carotene was used as a continuous variable with 3 knots (10th, 50th, and 90th percentiles), suggested by Harrell. Using the likelihood ratio test to examine nonlinearity, we compared models with only linear terms to those with both linear terms and cubic spline terms. Interaction and stratified analyses were implemented according to subgroup variables. Interaction across subgroups was tested using the likelihood ratio test. Missing data accounted for less than 20% of the data set and were handled by list wise deletion on an analysis basis. A set of sensitivity analyses was performed to assess the robustness of the study findings and to examine how different association inference models might influence our conclusions. The effect sizes and *p*-values computed from all these models were documented and compared. Analysis was conducted using R Statistical Software (Version 4.2.2, The R Foundation) and the Free Statistics analysis platform (Version 1.9, Beijing, China).

## 3 Results

### 3.1 Baseline characteristics

After rigorous screening based on the inclusion and exclusion criteria, the analysis included a total of 16,852 patients. Among them, the overall prevalence of PD disease was 1.82%, with 43.88% in males, and 56.12% in females. The weighted sample represents 8267.53 ten thousand people in the US (Table 1). The table shows the weighted numbers for each group. In this study, *β*-carotene levels were divided into three categories: tertile (T)1 (0–0.424 mg/d), T2 (0.425–1.660 mg/d), and T3 (1.661–203.516 mg/d), based on tertiles.

**Table 1:** The base line characteristics by tertiles of the *β*-Carotene: National Health and Nutrition Examination Survey 2001–2018, weighted.

Characteristics	Dietary *β*-carotene intake (mg/d)				
	Total(weighted)	T1 (0–0.422)	T2 (0.423–1.656)	T3 (1.657–203.516)	*p*- value
NO (ten thousand)	8267.53	2514.20	2817.23	2936.10	
Age (year), Mean ± SD	59.62 ± 11.77	59.00 ± 11.85	59.37 ± 11.92	60.39 ± 11.51	<0.0001
Sex, *n* (%)					
Male	3627.95 (43.88)	1062.55 (42.26)	1295.33 (45.98)	1270.07 (43.26)	0.0111
Female	4639.58 (56.12)	1451.65 (57.74)	1521.89 (54.02)	1666.03 (56.74)	
Race/ethnicity, *n* (%)					
Non-Hispanic White	6461.52 (78.16)	1891.51 (75.23)	2245.14 (79.69)	2324.86 (79.18)	<0.0001
Non-Hispanic Black	768.42 (9.29)	308.27 (12.26)	218.32 (7.75)	241.84 (8.24)	
Mexican American	345.66 (4.18)	107.29 (4.27)	134.07 (4.76)	104.31 (3.55)	
Other Hispanic	272.14 (3.29)	97.76 (3.89)	93.39 (3.31)	80.99 (2.76)	
Other Race	419.78 (5.08)	109.37 (4.35)	126.30 (4.48)	184.11 (6.27)	
Marital status, *n* (%)					
Married/ Living with a partner	5540.76 (67.02)	1589.98 (63.24)	1915.70 (68.00)	2035.08 (69.31)	<0.0001
Living alone	2726.77 (32.98)	924.22 (36.76)	901.52 (32.00)	901.02 (30.69)	
Family income, *n* (%)					
≤1.30	1391.45 (16.83)	585.06 (23.27)	428.96 (15.23)	377.43 (12.85)	<0.0001
1.31–3.50	2840.60 (34.36)	931.40 (37.05)	989.13 (35.11)	920.07 (31.34)	
>3.50	4035.47 (48.81)	997.74 (39.68)	1399.13 (49.66)	1638.60 (55.81)	
Education level, *n* (%)					
Less than high school	1262.84 (15.27)	525.26 (20.89)	396.51 (14.07)	341.07 (11.62)	<0.0001
High school or equivalent	2034.00 (24.60)	742.13 (29.52)	684.25 (24.29)	607.63 (20.70)	
Above high school	4970.69 (60.12)	1246.82 (49.59)	1736.47 (61.64)	1987.40 (67.69)	
BMI(kg/m^**2**^), Mean ± SD	29.75 ± 6.75	30.16 ± 7.00	29.90 ± 6.49	29.24 ± 6.73	<0.0001
Smoking status, *n* (%)					
Never	4073.51 (49.27)	1163.54 (46.28)	1394.57 (49.50)	1515.40 (51.61)	<0.0001
Former	2829.25 (34.22)	772.26 (30.72)	972.12 (34.51)	1084.87 (36.95)	
Current	1364.76 (16.51)	578.40 (23.01)	450.53 (15.99)	335.83 (11.44)	
Drinking status, *n* (%)					
Never	924.74 (11.19)	316.48 (12.59)	296.21 (10.51)	312.05 (10.63)	<0.0001
Former	1629.76 (19.71)	587.94 (23.38)	548.97 (19.49)	492.85 (16.79)	
Current	5713.03 (69.10)	1609.78 (64.03)	1972.05 (70.00)	2131.20 (72.59)	
Coronary heart disease, *n* (%)					
No	7660.14 (92.65)	2321.94 (92.35)	2596.05 (92.15)	2742.15 (93.39)	0.0912
Yes	607.39 (7.35)	192.26 (7.65)	221.17 (7.85)	193.95 (6.61)	
Stroke, *n* (%)					
No	7818.03 (94.56)	2347.02 (93.35)	2675.41 (94.97)	2795.59 (95.21)	0.0005
Yes	449.50 (5.44)	167.18 (6.65)	141.81 (5.03)	140.51 (4.79)	
Hypertension, *n* (%)					
No	3233.80 (39.11)	969.34 (38.55)	1090.05 (38.69)	1174.41 (40.00)	0.4799
Yes	5033.73 (60.89)	1544.86 (61.45)	1727.18 (61.31)	1761.69 (60.00)	
Diabetes, *n* (%)					
No	6328.07 (76.54)	1906.28 (75.82)	2139.22 (75.93)	2282.57 (77.74)	0.1511
Yes	1939.46 (23.46)	607.92 (24.18)	678.00 (24.07)	653.53 (22.26)	
Parkinson, *n* (%)					
No	8121.35 (98.23)	2453.71 (97.59)	2778.06 (98.61)	2889.58 (98.42)	0.0089
Yes	146.18 (1.77)	60.49 (2.41)	39.17 (1.39)	46.53 (1.58)	
All-cause mortality					
No	6787.52 (82.10)	2011.08 (79.99)	2310.57 (82.02)	2465.86 (83.98)	0.0001
Yes	1480.01 (17.90)	503.12 (20.01)	506.65 (17.98)	470.24 (16.02)	

T, tertiles; SD, standard deviation; BMI: body mass index.

Participants in the highest *β*-carotene tertile tended to be older, comprised more females, had a higher proportion of non-Hispanic whites, were more likely to be married or living with a partner, possessed greater wealth, attained higher levels of education, had lower BMI values, were fewer current smokers, were more current drinkers, had a lower prevalence of stroke and PD, had a lower all-cause mortality, in contrast to those in the lowest *β*-carotene tertile (all *p* value <0.05; Table 1).

### 3.2 Relationship between dietary *β*-carotene intake and PD

Univariate weighted logistic regression analysis revealed connections between PD and variables including family income, smoking status, stroke and dietary *β*-carotene intake (all *p* value <0.05; Supplementary Table S1).

In a multivariable weighted logistic regression analysis, after examining dietary beta-carotene intake as a continuous variable and adjusting for potential confounders, a significant negative linear correlation was found between dietary *β*-carotene intake and PD (OR = 0.95; 95% CI: 0.90 ~ 0.997; *p* = 0.040). This indicated that for every unit (mg/d) increase in dietary *β*-carotene intake, the prevalence of PD decreases by 6%.

**Figure 2:**
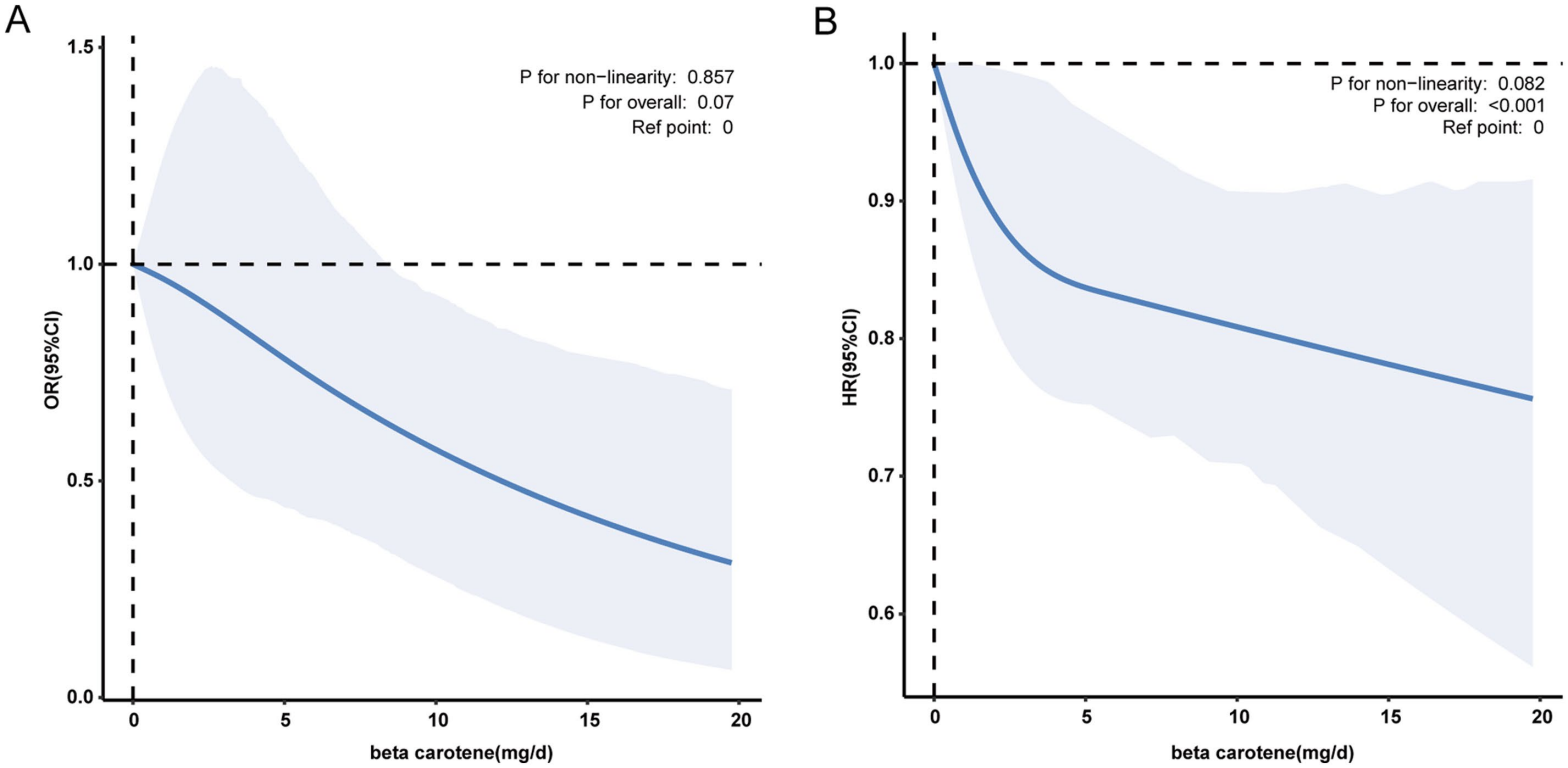
**(A)** The association between dietary *β*-carotene intake and the odds ratio of parkinson’s disease after multiple imputation. **(B)** The association between dietary *β*-carotene intake and the odds ratio of all-cause mortality after multiple imputation. Solid and dashed linesrepresent the predicted value and 95% confidence intervals. They were adjusted for age, sex, race/ethnicity, marital status, family income, education level, body mass index, smoking status, drinking status, coronary heart disease, stroke, hypertension and diabetes. Only 99% of the data is shown. Exegesis: OR, odds ratio; CI, confidence interval; HR, Hazard Ratio; Ref, Reference.

In contrast to individuals in the lowest dietary *β*-carotene intake tertile (T1), those in the middle (T2) and highest (T3) tertiles demonstrated adjusted ORs for PD of 0.62 (95% CI = 0.44 ~ 0.87) and 0.74 (95% CI = 0.49 ~ 1.12), respectively (Table 2). These results indicated a respective reduction of 38 and 26% in the risk of PD when comparing T2 and T3 to T1. Moreover, a RCS was utilized to estimate the possible dose–response connection between the dietary beta-carotene intake and the prevalence of PD. After accounting for confounding variables, an observation of a linear connection between dietary *β*-carotene intake and PD was made (*p* for nonlinearity = 0.857, Figure 2A).

**Table 2:** Associations between dietary *β*-carotene intake and Parkinson’s disease, weighted.

Variable	Dietary *β*-carotene intake (mg/d)		*β*-carotene levels tertiles (mg/d)		
			T1(0–0.424)	T2(0.425–1.660)	T3(1.661–203.516)
	OR (95% CI)	*P_*value	OR(95% CI)	OR(95% CI)	OR(95% CI)
NO.(unweighted)	16,852	5,609	5,624	5,619	
Model 1	0.93 (0.88–0.99)	0.014	1 (Ref)	0.57 (0.40–0.81)	0.65 (0.44–0.97)
Model 2	0.93 (0.88–0.98)	0.013	1 (Ref)	0.57 (0.40–0.80)	0.64 (0.43–0.95)
Model 3	0.94 (0.89–0.99)	0.028	1 (Ref)	0.60 (0.43–0.84)	0.70 (0.47–1.05)
Model 4	0.94 (0.90–0.997)	0.040	1 (Ref)	0.61 (0.44–0.86)	0.74 (0.49–1.12)
Model 5	0.95 (0.90–0.997)	0.040	1 (Ref)	0.62 (0.44–0.87)	0.74 (0.49–1.12)

T, Tertiles; OR, odds ratio; CI, confidence interval; Ref, Reference. aModel 1: with no covariate adjustment. bModel 2: adjusted for age, sex, race/ethnicity. cModel 3: Model 2 + marital status, education level, family income. dModel 4: Model 3 + BMI, smoking status, drinking status. eModel 5: Model 4 + coronary heart disease, stroke, hypertension, diabetes.

### 3.3 Relationship between dietary *β*-carotene intake and all-cause mortality

Univariate weighted cox regression analysis revealed associations between all-cause mortality and variables including age, sex, race/ethnicity, marital status, family income, education level, BMI, smoking status, drinking status, coronary heart disease, stroke, hypertension, diabetes, and dietary *β*-carotene intake (all *p* value <0.05; Supplementary Table S2).

In a multivariable weighted cox regression analysis, after examining dietary *β*-carotene intake as a continuous variable and considering potential confounders, a significant negative linear correlation was found between dietary *β*-carotene intake and all-cause mortality (HR = 0.98; 95% CI: 0.97 ~ 0.99; *p* = 0.002). This indicated that for every unit (mg/d) increase in dietary *β*-carotene intake, the all-cause mortality decreases by 2%.

**Figure 3:**
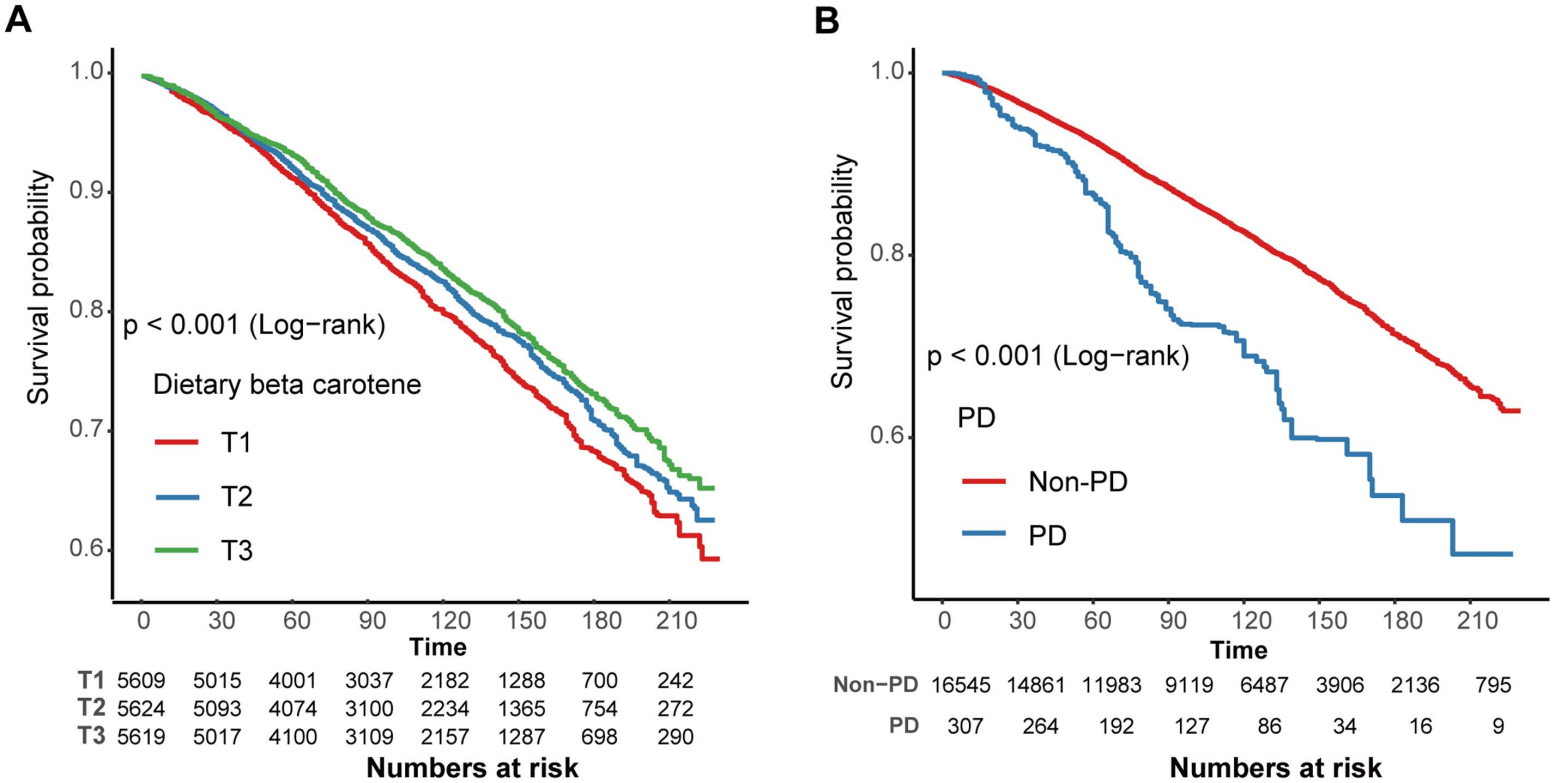
**(A)** Kaplan–Meier survival curve for dietary *β*-carotene intake and all-cause mortality. **(B)** Kaplan–Meier survival curve for Parkinson’s disease and all-cause mortality. Exegesis: T, Tertiles; PD, Parkinson’s disease.

In contrast to individuals in the lowest dietary *β*-carotene intake tertile (T1), those in the middle (T2) and highest (T3) tertiles demonstrated adjusted HRs for all-cause mortality of 0.93 (95% CI = 0.83 ~ 1.05) and 0.87 (95% CI = 0.79 ~ 0.96), respectively (Table 3). These results indicated a respective reduction of 7 and 13% in the all-cause mortality when comparing T2 and T3 to T1. Additionally, we plotted the KM curve, as shown in Figure 3A (log-rank test *p* < 0.001). Moreover, a RCS was utilized to estimate the possible dose–response connection between the dietary *β*-carotene intake and the prevalence of all-cause mortality. After accounting for confounding variables, an observation of a linear correlation between dietary *β*-carotene intake and all-cause mortality was made (*p* for nonlinearity = 0.082, Figure 2B).

**Table 3:** Associations between dietary *β*-carotene intake and all-cause mortality, weighted.

Variable	Dietary *β*-carotene intake (mg/d)		*β*-carotene levels tertiles (mg/d)		
			T1(0–0.424)	T2(0.425–1.660)	T3(1.661–203.516)
	HR(95% CI)	*p*- value	HR(95% CI)	HR(95% CI)	HR(95% CI)
NO.(unweighted)	16,852		5,609	5,624	5,619
Model 1	0.97 (0.96–0.99)	<0.001	1 (Ref)	0.89 (0.79–1.00)	0.82 (0.74–0.91)
Model 2	0.97 (0.96–0.98)	<0.001	1 (Ref)	0.84 (0.74–0.94)	0.72 (0.66–0.80)
Model 3	0.98 (0.97–0.99)	<0.001	1 (Ref)	0.90 (0.80–1.02)	0.81 (0.74–0.89)
Model 4	0.98 (0.97–0.99)	<0.001	1 (Ref)	0.93 (0.82–1.05)	0.86 (0.78–0.95)
Model 5	0.98 (0.97–0.99)	0.002	1 (Ref)	0.93 (0.83–1.05)	0.87 (0.79–0.96)

T, Tertiles; HR, Hazard Ratio; CI, confidence interval; Ref, Reference. aModel 1: with no covariate adjustment. bModel 2: adjusted for age, sex, race/Ethnicity. cModel 3: Model 2 + marital status, education level, family income. dModel 4: Model 3 + BMI, smoking status, drinking status. eModel 5: Model 4 + coronary heart disease, stroke, hypertension, diabetes.

### 3.4 Relationship between PD and all-cause mortality

After accounting for potential confounding factors, the adjusted HR for all-cause mortality in PD compared to non-PD individuals was 1.59 (95% CI: 1.22 ~ 2.06). These results indicate that the all-cause mortality rate in PD patients increased by 59% in comparison to non-PD individuals (Supplementary Table S3). Furthermore, PD showed a significant relation with all-cause mortality as demonstrated by the KM curves (log-rank test *p* < 0.001, Figure 3B).

### 3.5 Subgroup, sensitivity and additional analyses

**Figure 4:**
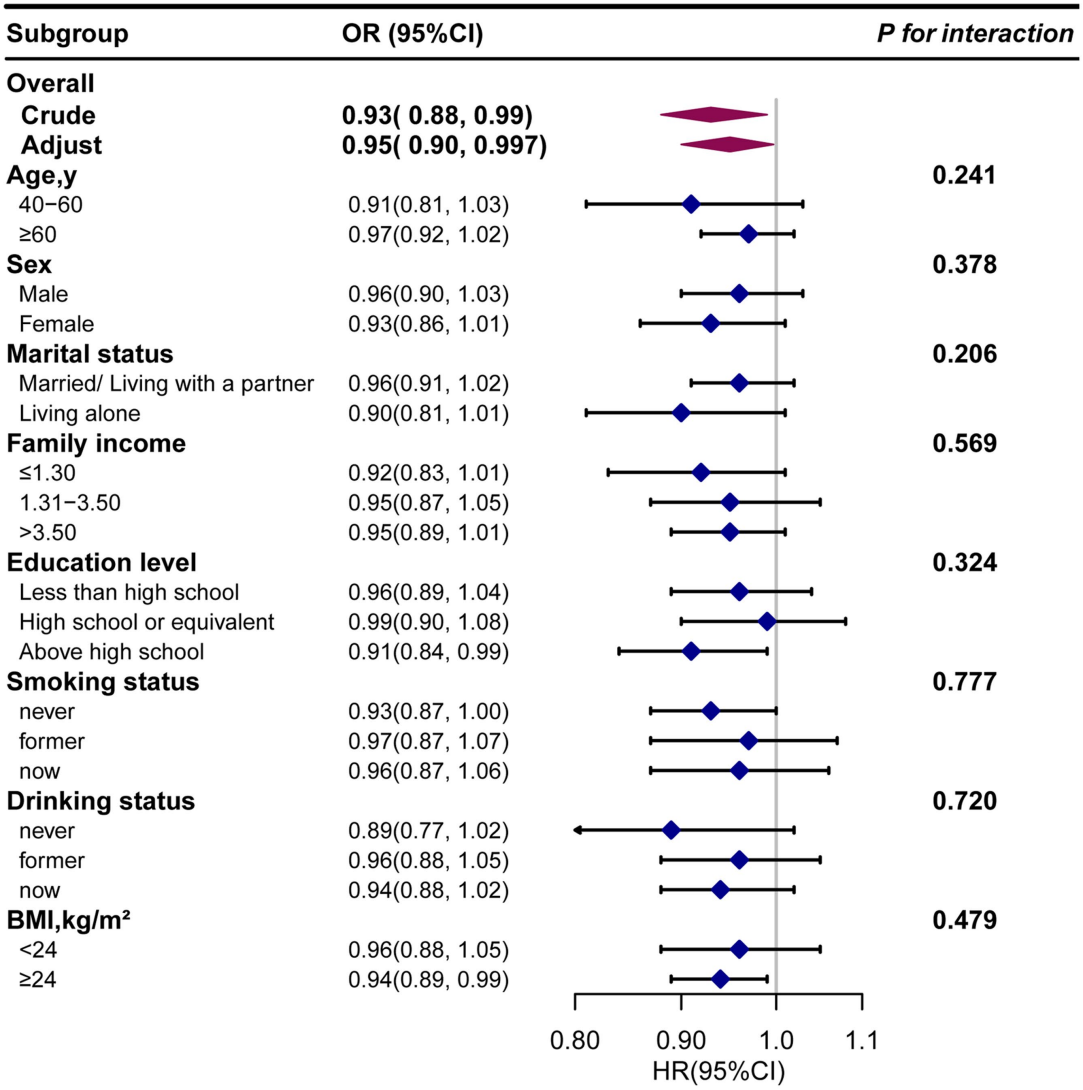
The relationship between the dietary *β*-carotene intake and Parkinson’s disease according to basic features. Except for the stratification component itself, each stratification factor was adjusted for all othervariables (age, sex, race/ethnicity, marital status, family income, education level, body mass index, smoking status, drinking status, coronary heart disease, stroke, hypertension and diabetes). Exegesis: OR, odds ratio; CI, confidence interval; BMI: body mass index.

**Figure 5:**
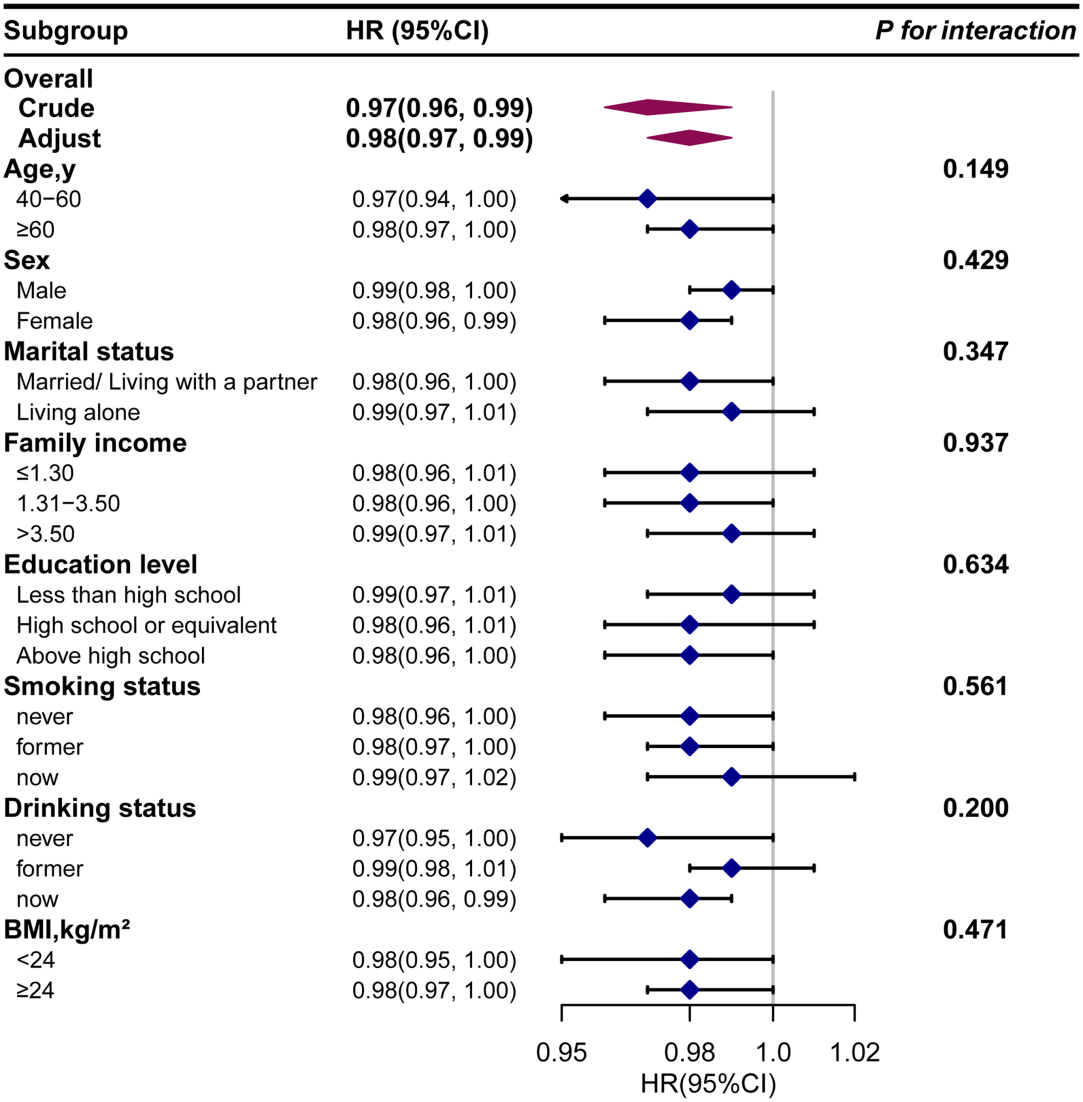
The relationship between the dietary *β*-carotene intake and all-cause mortality according to basic features. Except for the stratification component itself, each stratification factor was adjusted for all othervariables (age, sex, race/ethnicity, marital status, family income, education level, body mass index, smoking status, drinking status, coronary heart disease, stroke, hypertension and diabetes). Exegesis: HR, Hazard Ratio; CI, confidence interval; BMI: body mass index.

Next, we conducted subgroup analysis to evaluate potential modifiers on the relationship between dietary *β*-carotene intake and the prevalence of PD as well as all-cause mortality. Subgroups were stratified by age, sex, marital status, education level, family income, smoking status, drinking status, and BMI. However, no significant interactions were observed across any subgroup, indicating that these factors did not significantly modify the association between dietary *β*-carotene intake and the prevalence of PD or all-cause mortality (Supplementary Tables S4, S5; Figures 4, 5). In summary, the relationship between dietary *β*-carotene intake and PD as well as all-cause mortality was not significantly affected across different population subgroups.

For sensitivity analysis, we conducted five sets of multiple imputations and selected one dataset for multivariable analysis. In the meantime smoothed curve fitting was performed. Multifactorial analyses yielded results consistent with analyses that removed individuals with missing covariates (Supplementary Tables S6, S7).

Additionally, by reviewing the literature, we found that dietary vitamin E intake (37), dietary vitamin C intake (38), dietary iron intake (38), dietary copper intake (39), and dietary niacin intake (40) are associated with PD. Therefore, we included them as covariates for further analysis, and the results were consistent with the aforementioned findings (Supplementary Tables S8, S9).

In conclusion, the relationship between dietary *β*-carotene intake and both PD and all-cause mortality appears to be relatively robust.

## 4 Discussion

As far as we are aware, in this comprehensive retrospective cross-sectional study utilizing large NHANES datasets from 2001 to 2018, we consistently found an inverse correlation between dietary *β*-carotene intake and the prevalence of PD. Notably, these results remained consistent across various clinical subgroups and in sensitivity analyses. These findings carry significant clinical implications.

Until now, there have been few clinical reports on the correlation between dietary *β*-carotene intake and PD. In their comprehensive review, Wu et al. analyzed data from 4 cohort studies, 6 case–control studies, and 1 cross-sectional study, all of which consistently indicated a notably diminished risk of PD associated with higher *β*-carotene intake compared to lower intake levels (41). Additionally, a systematic review and meta-analysis confirmed the potential benefits of *β*-carotene intake in reducing PD risk, suggesting that a daily increase of 2 mg of *β*-carotene could contribute to risk reduction (15). A study involving 38,937 women and 45,837 men demonstrated an correlation between dietary *β*-carotene intake and a reduced risk of PD. (17) What’s more, a study conducted in South Korea further bolstered these findings by examining 104 patients diagnosed with idiopathic PD, matched with 52 healthy controls based on age and gender (42). The study revealed that PD patients exhibited lower serum levels of *β*-carotene compared to the control group (42). Moreover, within the PD patient group, those in advanced stages of the disease showed significantly lower *β*-carotene levels compared to those in early stages (42).

Furthermore, literature reviews have revealed that *β*-carotene may influence the progression of PD through the following mechanisms. *β*-carotene, proposed as a natural antioxidant, possesses the capability to capture and neutralize free radicals, and it has scavenging and quenching properties, thereby preventing oxidative stress (43–45). Recent research has underscored the pivotal role of *β*-carotene in combating oxidative stress and lipid peroxidation, thus establishing its significance in brain-related conditions (20, 46). Treatment with *β*-carotene has been found to enhance the preservation of intracellular antioxidants like glutathione and superoxide dismutase, while also modulating the Nrf2/Keap1 pathway, ultimately exerting neuroprotective effects (21). Moreover, nanoparticles incorporating *β*-carotene have shown promise in safeguarding against neuromotor damage, oxidative stress, and dopamine deficits in models of PD. These nanoparticles exhibit notable neuroprotective effects against Parkinson-like pathology, indicating their potential as a therapeutic intervention. Additionally, *β*-carotene supplementation has been associated with increased levels of neurotrophic factors, as evidenced by recent studies in mice (47, 48). Furthermore, in the context of aging, *β*-carotene has been implicated in mitigating age-related processes. Research suggests that *β*-carotene may delay the senescence of bone marrow mesenchymal stem cells by modulating the KAT7-P15 signaling axis, highlighting its potential as a significant anti-aging molecule (49). These findings collectively underscore the importance of *β*-carotene in the context of PD prevention and management strategies. Therefore, research on *β*-carotene may contribute to exploring new avenues for PD treatment and providing more comprehensive protection for neurological health.

Through the cohort study, we have identified a significant negative correlation between dietary *β*-carotene intake and all-cause mortality. However, there are very few reports on the association between dietary *β*-carotene intake with all-cause mortality. Some articles focus on the association between dietary *β*-carotene intake and serum *β*-carotene with other mortality. Two systematic reviews and meta-analyses have indicated that *β*-carotene supplementation may increase overall mortality rates (50, 51). But, another study, in a random-effects meta-analysis of all 31 trials, found that *β*-carotene supplements had no preventive effect on mortality (52). In addition, research conducted on the Japanese population indicated a notable inverse correlation between *β*-carotene dietary intake and the risk of mortality related to cardiovascular diseases, coronary heart disease, and other cardiovascular conditions (53). Research also indicates that increased dietary intake of *β*-carotene before diagnosis is linked to enhanced overall survival and specific survival in hepatocellular carcinoma (54). Another study indicated that a higher biochemical status of *β*-carotene was associated with lower overall mortality, as well as lower mortality from cardiovascular disease, heart disease, stroke, cancer, and other causes (55). Therefore, the effect of dietary *β*-carotene intake on mortality is not uniform in current research. Firstly, this may be related to factors such as the study design, the population studied, sample size, types and doses of *β*-carotene. Secondly, some studies suggest that synthetic *β*-carotene has different effects compared to natural *β*-carotene; for example, synthetic *β*-carotene may promote cancer formation. In some studies, patients may have used synthetic rather than natural *β*-carotene, which could influence the experimental results (56). So, more rigorous experimental designs should be developed to address these factors in future research.

Our study possesses several strengths. Initially, complex sampling weights and sample design were taken into account, utilizing a representative sample of the US population from NHANES, ensuring robustness and generalizability to a broader population. Secondly, rigorous control for confounding variables, including sociodemographic factors and relevant medical history, was conducted. Additionally, subgroup and sensitivity analyses were performed, yielding consistent findings.

This research also has some limitations. Firstly, the causal connection between dietary *β*-carotene intake and PD is difficult to determine on account of the cross-sectional nature of this study. Secondly, due to the nature of the NHANES database, diagnosing PD patients solely based on the use of anti-Parkinsonian medication may lead to potential misdiagnosis. Thirdly, the specific course of the disease and the type of medication given and the amount of medication taken and the course of medication taken were also variable in our study. Finally, potential confounding factors may still exist despite our consideration of various aspects of confounding.

## 5 Conclusion

The cross-sectional and cohort study reveals a significant negative correlation between dietary *β*-carotene intake and both the prevalence of PD and all-cause mortality in the general population. This suggested that supplementing with dietary *β*-carotene might have certain benefits for reducing the prevalence of PD and all-cause mortality. However, further rigorously designed expected studies are needed to establish the causal relationship between them.

## Data Availability

Publicly available datasets were analyzed in this study. This data can be found at: https://wwwn.cdc.gov/nchs/nhanes/.
