# Supplementary material for: Association between dietary β-carotene intake with Parkinson’s disease and all-cause mortality among American adults aged 40 and older (NHANES 2001–2018)
Source: Front Nutr. 2024 Sep 30;11:1430605. doi: 10.3389/fnut.2024.1430605 (PMC11472250; doi:10.3389/fnut.2024.1430605)
Supplement: Supplementary file 1 [file Table_1.DOC]

Supplementary Table 1 Association of covariates with parkinson’s disease, weighted.

| **Variable** | **OR (95%CI)** | ***P*_value** | **Variable** | **OR (95%CI)** | ***P*_value** |
| --- | --- | --- | --- | --- | --- |
| **Age (years)** | 1.01 (1.00-1.03) | 0.100 | **Smoking status** |  |  |
| **Sex** |  |  | never | 1 (Ref) |  |
| Male | 1 (Ref) |  | former | 0.66 (0.46-0.94) | 0.022 |
| Female | 1.04 (0.76-1.43) | 0.799 | now | 1.27 (0.83-1.95) | 0.263 |
| **Race/ethnicity** |  |  | **Drinking status** |  |  |
| Non-Hispanic White | 1 (Ref) |  | never | 1 (Ref) |  |
| Non-Hispanic Black | 0.88 (0.56-1.40) | 0.593 | former | 1.23 (0.77-1.96) | 0.378 |
| Mexican American | 0.85 (0.54-1.34) | 0.483 | now | 0.76 (0.47-1.24) | 0.267 |
| Other Hispanic | 1.06 (0.47-2.35) | 0.895 | **Coronary heart disease** |  |  |
| Other Race | 0.75 (0.35-1.63) | 0.472 | No | 1 (Ref) |  |
| **Marital status** |  |  | Yes | 1.06 (0.59-1.91) | 0.851 |
| Married/ Living with a partner | 1 (Ref) |  | **Stroke** |  |  |
| Living alone | 1.29(0.91-1.84) | 0.151 | No | 1 (Ref) |  |
| **Family income** |  |  | Yes | 2.68 (1.72-4.18) | <0.001 |
| ≤1.30 | 1 (Ref) |  | **Hypertension** | 1 (Ref) |  |
| 1.31-3.50 | 0.80 (0.59-1.10) | 0.165 | No |  |  |
| >3.50 | 0.53 (0.34-0.83) | 0.006 | Yes | 1.15 (0.85-1.55) | 0.379 |
| **Education level** |  |  | **Diabetes** |  |  |
| High school or less | 1 (Ref) |  | No | 1 (Ref) |  |
| Some College | 0.87 (0.54-1.39) | 0.550 | Yes | 1.04 (0.75-1.43) | 0.817 |
| College graduate | 0.70 (0.47-1.03) | 0.072 | β**-Carotene (mg/d)** | 0.93 (0.88-0.99) | 0.014 |
| **BMI (kg/m^2^)** | 1.01 (0.99-1.03) | 0.298 | **\** | \ | \ |

OR, odds ratio; CI, confidence interval; BMI, body mass index.

Supplementary Table 2 Association of covariates with all-cause mortality, weighted.

| **Variable** | **HR (95%CI)** | ***P*_value** | **Variable** | **HR (95%CI)** | ***P*_value** |
| --- | --- | --- | --- | --- | --- |
| **Age (years)** | 1.1 (1.09-1.10) | <0.001 | **Smoking status** |  |  |
| **Sex** |  |  | never | 1 (Ref) |  |
| Male | 1 (Ref) |  | former | 1.65 (1.50-1.81) | <0.001 |
| Female | 0.72 (0.67-0.78) | <0.001 | now | 1.49 (1.34-1.66) | <0.001 |
| **Race/ethnicity** |  |  | **Drinking status** |  |  |
| Non-Hispanic White | 1 (Ref) |  | never | 1 (Ref) |  |
| Non-Hispanic Black | 1.05 (0.93-1.18) | 0.416 | former | 1.18 (1.02-1.36) | 0.026 |
| Mexican American | 0.69 (0.58-0.83) | <0.001 | now | 0.52 (0.46-0.60) | <0.001 |
| Other Hispanic | 0.78 (0.60-1.00) | 0.05 | **Coronary heart disease** |  |  |
| Other Race | 0.69 (0.53-0.90) | 0.006 | No | 1 (Ref) |  |
| **Marital status** |  |  | Yes | 3 (2.64-3.41) | <0.001 |
| Married/ Living with a partner | 1 (Ref) |  | **Stroke** |  |  |
| Living alone | 1.93 (1.77-2.11) | <0.001 | No | 1 (Ref) |  |
| **Family income** |  |  | Yes | 3.25 (2.83-3.73) | <0.001 |
| ≤1.30 | 1 (Ref) |  | **Hypertension** | 1 (Ref) |  |
| 1.31-3.50 | 0.78 (0.71-0.86) | <0.001 | No |  |  |
| >3.50 | 0.33 (0.29-0.37) | <0.001 | Yes | 2.21 (1.99-2.46) | <0.001 |
| **Education level** |  |  | **Diabetes** |  |  |
| High school or less | 1 (Ref) |  | No | 1 (Ref) |  |
| Some College | 0.63 (0.56-0.71) | <0.001 | Yes | 2.03 (1.84-2.23) | <0.001 |
| College graduate | 0.4 (0.36-0.45) | <0.001 | β**-Carotene (mg/d)** | 0.97 (0.96-0.99) | <0.001 |
| **BMI (kg/m^2^)** | 0.99 (0.98-1.00) | 0.02 | **\** | \ | \ |

HR, Hazard Ratio; CI, confidence interval; Ref, Reference; BMI, body mass index.

Supplementary Table 3 Associations between parkinson’s disease and all-cause mortality, weighted.

| **Variable** | **Non-PD** | **PD** | ***P_*value** |
| --- | --- | --- | --- |
|  | **HR(95% CI)** | **HR(95% CI)** |  |
| **NO.(unweighted)** | 16545 | 307 |  |
| **Model 1** | 1 (Ref) | 1.93 (1.43,2.59) | <0.001 |
| **Model 2** | 1 (Ref) | 1.73 (1.34,2.23) | <0.001 |
| **Model 3** | 1 (Ref) | 1.63 (1.25,2.13) | <0.001 |
| **Model 4** | 1 (Ref) | 1.60 (1.22,2.09) | <0.001 |
| **Model 5** | 1 (Ref) | 1.59 (1.22,2.06) | <0.001 |

HR, Hazard ratio; CI, confidence interval; Ref, Reference.

aModel 1: with no covariate adjustment.

bModel 2: adjusted for age,sex, race/ethnicity.

cModel 3: Model 2+marital status, education level, family income.

dModel 4: Model 3+BMI, smoking status, drinking status.

eModel 5: Model 4+coronary heart disease, stroke, hypertension, diabetes.

**Supplementary Table 4 Subgroup analysis of the association between dietary β-carotene intake and parkinson’s disease, weighted.**

| **Subgroup** | **OR(95%CI)** | ***P*_value** | ***P* for interaction** |
| --- | --- | --- | --- |
| **Age, y** |  |  | 0.241 |
| 40-60 | 0.91(0.81-1.03) | 0.14 |  |
| ≥60 | 0.97(0.92-1.02) | 0.27 |  |
| **Sex** |  |  | 0.378 |
| Male | 0.96(0.90-1.03) | 0.25 |  |
| Female | 0.93(0.86-1.01) | 0.08 |  |
| **Marital status** |  |  | 0.206 |
| Married/ Living with a partner | 0.96(0.91-1.02) | 0.17 |  |
| Living alone | 0.90(0.81-1.01) | 0.08 |  |
| **Family income** |  |  | 0.569 |
| ≤1.30 | 0.92(0.83-1.01) | 0.09 |  |
| 1.31-3.50 | 0.95(0.87-1.05) | 0.33 |  |
| >3.50 | 0.95(0.89-1.01) | 0.09 |  |
| **Education level** |  |  | 0.324 |
| Less than high school | 0.96(0.89-1.04) | 0.35 |  |
| High school or equivalent | 0.99(0.90-1.08) | 0.79 |  |
| Above high school | 0.91(0.84-0.99) | 0.03 |  |
| **Smoking status** |  |  | 0.777 |
| never | 0.93(0.87-1.00) | 0.04 |  |
| former | 0.97(0.87-1.07) | 0.51 |  |
| now | 0.96(0.87-1.06) | 0.45 |  |
| **Drinking status** |  |  | 0.720 |
| never | 0.89(0.77-1.02) | 0.1 |  |
| former | 0.96(0.88-1.05) | 0.42 |  |
| now | 0.94(0.88-1.02) | 0.12 |  |
| **BMI, kg/m^2^** |  |  | 0.479 |
| <24 | 0.96(0.88-1.05) | 0.4 |  |
| ≥24 | 0.94(0.89-0.99) | 0.02 |  |

OR, odds ratio; CI, confidence interval; BMI, body mass index.

**Supplementary Table 5 Subgroup analysis of the association between dietary β-carotene intake and all-cause mortality, weighted.**

| **Subgroup** | **HR(95%CI)** | ***P*_value** | ***P* for interaction** |
| --- | --- | --- | --- |
| **Age, y** |  |  | 0.149 |
| 40-60 | 0.97(0.94-1.00) | 0.04 |  |
| ≥60 | 0.98(0.97-1.00) | 0.01 |  |
| **Sex** |  |  | 0.429 |
| Male | 0.99(0.98-1.00) | 0.08 |  |
| Female | 0.98(0.96-0.99) | 0.01 |  |
| **Marital status** |  |  | 0.347 |
| Married/ Living with a partner | 0.98(0.96-1.00) | 0.01 |  |
| Living alone | 0.99(0.97-1.01) | 0.21 |  |
| **Family income** |  |  | 0.937 |
| ≤1.30 | 0.98(0.96-1.01) | 0.14 |  |
| 1.31-3.50 | 0.98(0.96-1.00) | 0.01 |  |
| >3.50 | 0.99(0.97-1.01) | 0.22 |  |
| **Education level** |  |  | 0.634 |
| Less than high school | 0.99(0.97-1.01) | 0.33 |  |
| High school or equivalent | 0.98(0.96-1.01) | 0.18 |  |
| Above high school | 0.98(0.96-1.00) | 0.02 |  |
| **Smoking status** |  |  | 0.561 |
| never | 0.98(0.96-1.00) | 0.02 |  |
| former | 0.98(0.97-1.00) | 0.07 |  |
| now | 0.99(0.97-1.02) | 0.63 |  |
| **Drinking status** |  |  | 0.200 |
| never | 0.97(0.95-1.00) | 0.06 |  |
| former | 0.99(0.98-1.01) | 0.56 |  |
| now | 0.98(0.96-0.99) | 0.01 |  |
| **BMI, kg/m^2^** |  |  | 0.471 |
| <24 | 0.98(0.95-1.00) | 0.08 |  |
| ≥24 | 0.98(0.97-1.00) | 0.02 |  |

HR, Hazard ratio; CI, confidence interval; BMI, body mass index.

**Supplementary Table 6** Associations between dietary β-carotene intake and parkinson’s disease (multiple interpolation).

| **Variable** | **Dietary β-carotene intake** | | **β-carotene levels tertiles (mg/d)** | | | | |
| --- | --- | --- | --- | --- | --- | --- | --- |
|  |  |  | **T1(0-0.418)** |  | **T2(0.419-1.655)** |  | **T3(1.656-203.516)** |
|  | **OR(95% CI)** | ***P_*value** | **OR(95% CI)** |  | **OR(95% CI)** |  | **OR(95% CI)** |
| **NO.(unweighted)** | 20389 | | 6794 |  | 6795 |  | 6800 |
| **Model 1** | 0.92 (0.87-0.97) | 0.001 | 1 (Ref) |  | 0.60 (0.44-0.82) |  | 0.63 (0.45-0.88) |
| **Model 2** | 0.91 (0.87-0.96) | 0.001 | 1 (Ref) |  | 0.59 (0.44-0.81) |  | 0.62 (0.45-0.85) |
| **Model 3** | 0.93 (0.88-0.97) | 0.003 | 1 (Ref) |  | 0.63 (0.46-0.86) |  | 0.68 (0.49-0.95) |
| **Model 4** | 0.93 (0.89-0.98) | 0.005 | 1 (Ref) |  | 0.64 (0.47-0.88) |  | 0.72 (0.51-1.00) |
| **Model 5** | 0.93 (0.89-0.98) | 0.002 | 1 (Ref) |  | 0.65 (0.48-0.89) |  | 0.73 (0.52-1.02) |

T, Tertiles; OR, odds ratio; CI, confidence interval; Ref, Reference.

aModel 1: with no covariate adjustment.

bModel 2: adjusted for age,sex, race/ethnicity.

cModel 3: Model 2+marital status, education level, family income.

dModel 4: Model 3+BMI, smoking status, drinking status.

eModel 5: Model 4+coronary heart disease, stroke, hypertension, diabetes.

**Supplementary Table 7** Associations between dietary β-carotene intake and all-cause mortality (multiple interpolation).

| **Variable** | **Dietary β-carotene intake (mg/d)** | | **β-carotene levels tertiles (mg/d)** | | | | |
| --- | --- | --- | --- | --- | --- | --- | --- |
|  |  |  | **T1(0-0.424)** |  | **T2(0.425-1.660)** |  | **T3(1.661-203.516)** |
|  | **HR(95% CI)** | ***P_*value** | **HR(95% CI)** |  | **HR(95% CI)** |  | **HR(95% CI)** |
| **NO.(unweighted)** | 20389 | | 6794 |  | 6795 |  | 6800 |
| **Model 1** | 0.98 (0.97-0.99) | <0.001 | 1 (Ref) |  | 0.89 (0.80, 0.99) |  | 0.82 (0.75, 0.90) |
| **Model 2** | 0.97 (0.96-0.98) | <0.001 | 1 (Ref) |  | 0.81 (0.74-0.90) |  | 0.71 (0.65-0.77) |
| **Model 3** | 0.98 (0.97-0.99) | <0.001 | 1 (Ref) |  | 0.88 (0.79-0.97) |  | 0.79 (0.73-0.87) |
| **Model 4** | 0.99 (0.98-1.00) | 0.011 | 1 (Ref) |  | 0.91 (0.82-1.01) |  | 0.85 (0.78-0.93) |
| **Model 5** | 0.99 (0.98-1.00) | 0.026 | 1 (Ref) |  | 0.91 (0.82-1.01) |  | 0.87 (0.79-0.94) |

T, Tertiles; HR, Hazard Ratio; CI, confidence interval; Ref, Reference.

aModel 1: with no covariate adjustment.

bModel 2: adjusted for age, sex, race/ethnicity.

cModel 3: Model 2+marital status, education level, family income.

dModel 4: Model 3+BMI, smoking status, drinking status.

eModel 5: Model 4+coronary heart disease, stroke, hypertension, diabetes.

**Supplementary Table 8** Associations between dietary β-carotene intake and parkinson’s disease (add other dietary markers).

| **Variable** | **Dietary β-carotene intake** | | **β-carotene levels tertiles (mg/d)** | | | | |
| --- | --- | --- | --- | --- | --- | --- | --- |
|  |  |  | **T1(0-0.424)** |  | **T2(0.425-1.660)** |  | **T3(1.661-203.516)** |
|  | **OR(95% CI)** | ***P_*value** | **OR(95% CI)** |  | **OR(95% CI)** |  | **OR(95% CI)** |
| **NO.(unweighted)** | 16852 | | 5609 |  | 5624 |  | 5619 |
| **Crude Model** | 0.93 (0.88-0.99) | 0.014 | 1 (Ref) |  | 0.57 (0.40-0.81) |  | 0.65 (0.44-0.97) |
| **^a^Adjusted Model** | 0.95 (0.90-0.99) | 0.028 | 1 (Ref) |  | 0.65 (0.45-0.93) |  | 0.78 (0.53-1.15) |

T, Tertiles; OR, odds ratio; CI, confidence interval; Ref, Reference.

**^a^Adjusted Model**: Adjusted for age, sex, race/ethnicity, marital status, family income, education level, body mass index, smoking status, drinking status, coronary heart disease, stroke, hypertension, diabetes, dietary vitamin E intake, dietary vitamin C intake, dietary copper intake, dietary iron intake and dietary niacin intake.

**Supplementary Table 9** Associations between dietary β-carotene intake and all-cause mortality (add other dietary markers).

| **Variable** | **^a^Dietary β-carotene intake** | | **β-carotene levels tertiles (mg/d)** | | | | |
| --- | --- | --- | --- | --- | --- | --- | --- |
|  |  |  | **T1(0-0.424)** |  | **T2(0.425-1.660)** |  | **T3(1.661-203.516)** |
|  | **HR(95% CI)** | ***P_*value** | **HR(95% CI)** |  | **HR(95% CI)** |  | **HR(95% CI)** |
| **NO.(unweighted)** | 16852 | | 5609 |  | 5624 |  | 5619 |
| **Crude Model** | 0.78 (0.69-0.87) | <0.001 | 1 (Ref) |  | 0.89 (0.79-1.00) |  | 0.82 (0.74-0.91) |
| **^b^Adjusted Model** | 0.86 0.77-0.97) | 0.011 | 1 (Ref) |  | 0.95 (0.84-1.07) |  | 0.89 (0.80-0.99) |

T, Tertiles; HR, Hazard Ratio; CI, confidence interval; Ref, Reference.

^a^Dietary β-carotene intake was treated as a continuous variable and multiplied by 0.1 for a multiplicative transformation.

**^b^Adjusted Model**: Adjusted for age, sex, race/ethnicity, marital status, family income, education level, body mass index, smoking status, drinking status, coronary heart disease, stroke, hypertension, diabetes, dietary vitamin E intake, dietary vitamin C intake, dietary copper intake, dietary iron intake and dietary niacin intake.
